# Supplementary material for: In silico design of novel recombinant antigens containing immunologically relevant regions of wild-type and escape mutant variants of HBsAg
Source: PLoS One. 2026 Mar 9;21(3):e0344362. doi: 10.1371/journal.pone.0344362 (PMC12970925; doi:10.1371/journal.pone.0344362)
Supplement: S1 File — (PDF) [file pone.0344362.s001.pdf]

S1 Table. Frequencies of EMAVs in the MHR of HBV genotypes A and D circulating in Ethiopia.

Genotype A				Genotype D			
Mutation	Frequency	Mutation	Frequency	Mutation	Frequency	Mutation	Frequency
Y161F	28	P120L	1	P127T	92	Q101H	1
F134L	7	T114I	1	T118A	85	L104W	1
G130N	6	T114K	1	S143L	15	L109Q	1
E164D	6	L109I	1	R122K	9	L109M	1
T118A	6	V106G	1	Q129H	7	T118S	1
Q129R	5	S113T	1	G159A	7	S132Y	1
F134Y	5	G102H	1	T131N	5	L104S	1
I110L	4	M103T	1	Y134F	4	S113T	1
A159V	4	I118M	1	I110L	4	Y134S	1
E164G	4	T116N	1	Y134N	4	F158L	1
N131T	4	T125I	1	M103I	3	A166V	1
T140I	3	S136I	1	A128V	3	S143M	1
G119R	3	T126N	1	P120S	3	K160N	1
P135H	3	F134V	1	P120T	3	E164G	1
M133T	3	T125S	1	D144E	3	E164V	1
T143M	3	C137S	1	A168V	3	A168P	1
V168A	3	P127T	1	S114T	2	G159E	1
T126I	3	I150T	1	M133I	2	K160S	1
T114S	2	W165S	1	S114P	2	E164D	1
M103I	2	V168A	1	F161Y	2	I150F	1
L109P	2	L162H	1	R169H	2	P127A	1
T118P	2	R169A	1	S143T	2	P127L	1
G119E	2	K141I	1	P105V	1		
Y100C	2	K160R	1	T118V	1		
P120T	2	S167L	1	T131P	1		
N131S	2	T148P	1	P135L	1		
M133I	2	W165R	1	G112N	1		
D144A	2	S155F	1	L104F	1		
K122R	2	K160N	1	M133V	1		
T143L	2	T143S	1	G102H	1		
L109Q	1	V159G	1	T131I	1		
P111N	1			Q101R	1		
