# Supplementary material for: In silico design of novel recombinant antigens containing immunologically relevant regions of wild-type and escape mutant variants of HBsAg
Source: PLoS One. 2026 Mar 9;21(3):e0344362. doi: 10.1371/journal.pone.0344362 (PMC12970925; doi:10.1371/journal.pone.0344362)
Supplement: Attachment — Submitted filename: ReviewersComments_1.22.26.pdf [file pone.0344362.s008.pdf]

Dear Authors,

This manuscript presents a comprehensive in silico design and characterization of three recombinant HBsAg-derived antigens (MeRPYS1–3) intended for improved HBV diagnostic applications in Ethiopia. The study integrates sequence epidemiology, epitope prediction, structural modeling, molecular docking, and molecular dynamics simulations.

The work addresses a relevant public-health and diagnostic problem, particularly in regions with high HBV burden and genotype diversity. The computational pipeline is extensive and technically competent. However, the manuscript currently overinterprets in silico findings, lacks clarity in several methodological justifications, and requires significant tightening of claims to meet PLOS ONE's standards for methodological rigor and reproducibility.

Comments (Should be addressed)

1. Overinterpretation of in silico predictions

While the authors repeatedly acknowledge the lack of experimental validation, several conclusions still go beyond what is justified by computational analyses alone.

Examples:

- Statements suggesting "stable antigen–antibody interactions" and "supporting candidacy for polyclonal antibody production" are not experimentally substantiated.
- MD simulation stability is interpreted as evidence of functional binding, which is not equivalent to biochemical affinity or immunoreactivity.

Recommendation:
Rephrase conclusions throughout the Results, Discussion, and Abstract to emphasize hypothesis-generating rather than confirmatory outcomes. Avoid language implying functional efficacy.

2. Limited novelty relative to existing in silico multi-epitope antigen studies

The manuscript claims to be the first to computationally design region-specific HBV antigens. However:

- Similar multi-epitope and mutation-inclusive HBsAg designs have been reported previously (including outside Ethiopia).
- The novelty here appears to be regional sequence prioritization, not the computational strategy itself.

Recommendation:
Clarify novelty explicitly:

- Emphasize Ethiopia-specific EMAV prevalence data
- Avoid blanket "first study" claims unless clearly justified with citations

3. Statistical analysis is correct but conceptually underused

The genotype A vs D comparison is statistically strong ($z = 12.44$, $\Delta p = 50.7\%$), but:

- This result is not integrated mechanistically into antigen design decisions.
- It is unclear how genotype-specific mutation prevalence quantitatively informed MeRPYS1 vs MeRPYS2 composition.

Recommendation:
Explicitly link statistical findings to design rationale:

- Why do MeRPYS1 and MeRPYS2 differ?
- How does genotype D's higher EMAV burden influence antigen architecture?

4. Epitope prediction redundancy inflates perceived immunogenicity

The repeated duplication of identical or near-identical epitopes is presented as beneficial, but:

- Epitope repetition does not guarantee improved immunogenicity
- Repetition can cause epitope masking, misfolding, or immune tolerance

The manuscript mentions this limitation briefly, but still implicitly treats duplication as advantageous.

Recommendation:
Strengthen critical discussion:

- Explicitly state that epitope duplication is speculative
- Cite studies where repetition failed or reduced immunogenicity

5. Molecular docking and MD simulation limitations are underemphasized

Concerns:

- Only one antibody structure (PDB: 6VJT) was used
- MD simulations were run once per complex, without replicas
- Binding free energy was not calculated (e.g., MM-PBSA/MM-GBSA)

Recommendation:
Either:

- Add justification for these limitations
  or
- Explicitly acknowledge that docking + MD are qualitative, not quantitative

6. Diagnostic relevance remains hypothetical

The manuscript repeatedly frames the antigens as candidates for diagnostic assay development, but:

- No comparison is made to existing commercial HBsAg antigens
- No sensitivity/specificity modeling is attempted

Recommendation:
Tone down diagnostic claims or add a short section outlining:

- How these antigens would outperform current kits
- What specific diagnostic formats are envisioned (ELISA, lateral flow, etc.)

Minor Comments (Should be addressed)

1. Language and clarity
   - Numerous grammatical errors (e.g., "excremental validation" → experimental validation)
   - Inconsistent spacing, punctuation, and hyphenation
2. Figure density
   - Some figures (especially MD plots) are excessive for PLOS ONE
   - Consider moving secondary MD plots to Supporting Information
3. Methods reproducibility
   - Random seed handling in MD simulations should be more clearly described
   - Provide exact software versions consistently (some are missing)
4. Ethics statement
   - Although sequence data are public, explicitly state that no new human data were generated
5. References
   - Some citations supporting epitope prediction accuracy are outdated
   - Consider adding recent benchmarking studies (2022–2024)

Overall Recommendation

Minor Revision

Rationale:
The study is technically solid, regionally relevant, and suitable for PLOS ONE if claims are appropriately restrained and methodological justifications strengthened. The manuscript currently reads closer to a computational validation paper than a diagnostic development study, and this distinction must be made explicit.
