# Supplementary material for: In silico design of novel recombinant antigens containing immunologically relevant regions of wild-type and escape mutant variants of HBsAg
Source: PLoS One. 2026 Mar 9;21(3):e0344362. doi: 10.1371/journal.pone.0344362 (PMC12970925; doi:10.1371/journal.pone.0344362)
Supplement: Attachment — Submitted filename: PONE_D_25_38657_Reviewed by SB_8.16.25.docx [file pone.0344362.s003.docx]

**Review for “In silico design of novel recombinant antigens containing immunologically relevant regions of wild-type and escape mutant variants of HBsAg”**

**I appreciate the hard work and the scientific approach. It’s a well written manuscript. I would like the authors to revise on the following points: I am adding my suggestions as well.**

1. ***Since the entire study is computational, with no experimental validation (e.g., expression, purification, serological testing). The conclusions about diagnostic utility are speculative.***

Authors can acknowledge this limitation clearly and, write a future plan to validate their designed constructs through:

- Cloning and expression in E. coli or mammalian cells.
- ELISA testing with sera from HBV-positive patients (wild-type and escape mutants).
- Stability/solubility checks of recombinant proteins.

2. ***The dataset includes only 617 sequences from Ethiopia, up to April 2021. This may not represent global or even current Ethiopian HBV diversity.***

- Expand the dataset to include global sequences from GenBank, especially genotypes B, C, and E which are common in Asia and Africa.
- Incorporate newer sequences (2022–2025) to ensure up-to-date coverage.
- Perform phylogenetic comparison to demonstrate broader relevance.

3. ***The study focuses only on linear B-cell epitopes, whereas HBV antigens are known to have conformational epitopes critical for antibody recognition.***

- Include conformational epitope prediction (e.g., DiscoTope, ElliPro).
- Consider T-cell epitope predictions to understand the immunogenic landscape more fully.
- Validate epitope conservancy against a larger HBV sequence dataset.

4. ***The recombinant designs (MeRPYS1–3) contain tandem repeats of the MHR and escape mutant regions linked by GGS linkers. Such designs may cause misfolding, aggregation, or reduced solubility in real expression systems.***

- Evaluate protein solubility and folding using in silico tools (e.g., SOLpro, Aggrescan).
- Explore shorter constructs or mosaic antigens instead of long repeats.
- Consider codon optimization for expression in the chosen system.

5. ***Structural validation relies mainly on Ramachandran plots and ProSA. Docking is performed with hNTCP, which is a receptor for viral entry, not relevant for diagnostic antibody binding.***

- Dock recombinant proteins against anti-HBsAg antibodies (available in PDB) instead of hNTCP, since the diagnostic purpose relates to antigen–antibody recognition.
- Complement I-TASSER results with AlphaFold2 predictions for higher structural confidence.

6. ***MD was done using I-MODS (normal mode analysis), which is a coarse approximation and not a full atomistic MD simulation.***

- Perform atomistic MD simulations (e.g., GROMACS, AMBER) with explicit solvent models to assess stability and conformational dynamics.
- Report RMSD, RMSF, Rg, and hydrogen-bond stability metrics.

7. ***The conclusion that these constructs are “promising candidate antigens” is overstated without experimental evidence.***

- Reframe conclusions to highlight that the study provides a computational framework for antigen design, and that further experimental validation is needed.
- Provide a more balanced discussion of limitations.

**In summary:**
The manuscript provides a valuable computational pipeline but lacks experimental depth. To improve, the authors should (i) broaden sequence coverage, (ii) include conformational epitope analysis, (iii) redesign docking studies with antibodies, (iv) apply more robust MD simulations, and (v) clearly state the need for laboratory validation.
