# Supplementary material for: In silico design of novel recombinant antigens containing immunologically relevant regions of wild-type and escape mutant variants of HBsAg
Source: PLoS One. 2026 Mar 9;21(3):e0344362. doi: 10.1371/journal.pone.0344362 (PMC12970925; doi:10.1371/journal.pone.0344362)
Supplement: Attachment — Submitted filename: Response to Reviewers.docx [file pone.0344362.s005.docx]

**Response to Reviewers’ Comments and Questions**

**Reviewer #1**

1. **Please elaborate on the quality control standards used for the 617 NCBI sequences in Section 2.1. Explain why April 2021 was selected as the cut-off date and whether geographic sampling bias could have an impact on genotype frequency results. The manuscript doesn't include how sequence quality was assessed and if low quality sequences were excluded? Also, please mention if duplicates were removed to avoid bias and if any filtering was done to include all sequences from Ethiopian-origin patients.**

*Thank you for your thoughtful feedback and careful review of our manuscript. We welcome the opportunity to clarify our data curation and quality-control workflow.*

***Cut-off date (April 2021):*** *This date marked the end of our original data-collection period and reflected the most recent sequences available when the study was initiated. The analysis has now been updated to include all sequences released through August 2025 (670 total), as described in the revised manuscript.*

***Quality control:*** *Our work differs from a typical next-generation sequencing (NGS) pipeline that starts with raw FASTQ reads requiring tools such as FastQC or Trimmomatic. Instead, we analyzed curated, annotated protein sequences deposited in NCBI, which have already undergone quality review by the submitting laboratories and GenBank curators. Conventional raw-data trimming and filtering are therefore not applicable.*

*However, since our focus is the HBsAg major hydrophilic region (MHR), particularly the “a” determinant, we included only Ethiopian isolates with complete amino-acid coverage in these regions. Each sequence was examined in BioEdit, and any isolate containing an “x” (unknown amino acid) in the “a” determinant was excluded. Using this criterion, 10 of 682 available Ethiopian HBsAg isolates were removed. One isolate was excluded as it was the only isolated classified as genotype E and the other one was excluded since the HBsAg amino acid was not uploaded. A total of 670 HBV isolates from Ethiopia are included in the data analysis.*

***Geographic sampling bias:*** *We recognize that sequence availability depends on local surveillance and reporting and may over-represent certain regions or collection periods. This limitation is now addressed in the limitation section.*

***Geographic and genotypic filtering:*** *To focus on immunologically relevant variants circulating in Ethiopia, we retained only sequences from Ethiopian patients and those belonging to genotypes A and D, which predominate in the country. This targeted filtering ensures our dataset reflects the strains of greatest clinical and diagnostic relevance rather than a random sample of global HBV diversity.*

2. **Please mention the database used, the E-value cutoff, the identity threshold, the coverage criteria, and the BLAST program (such as blastp) in Section 2.3.The term "no homology" is not defined; is it referring to a threshold of less than 30% identity over the entire sequence, or something else? The claim cannot be independently verified without this information, and the result is hard to replicate.**

*We thank the reviewer for their insightful questions, comments and suggestions. We appreciate it. This helped and encouraged us to enrich our analysis.*

*All three proteins (MeRPYS1–3) were analyzed using BLASTp (National Center for Biotechnology Information; https://blast.ncbi.nlm.nih.gov/Blast.cgi) against both human proteins (Homo sapiens, taxid:9606) and mouse proteins (Mus musculus Balb/c, taxid:10090). The searches were performed using the Reference Protein (RefSeq_protein) database, with an E-value cutoff of 0.05. A sequence identity greater than 35% combined with query coverage above 80% was considered indicative of potential similarity or cross-reactivity with host proteins.*

*The results demonstrated that none of the three recombinant proteins (MeRPYS1–3) showed significant similarity to either human or mouse proteins. They were non-homologous with human and mouse proteins.*

3. **Predictions in Section 2.4 are performed using only one tool (BCEPRED). Despite being a useful tool, BCEPRED has certain drawbacks that need to be addressed: using just one algorithm raises the possibility of false positives or negatives. There are no parameters provided, such as window size or the propensity scale. In the modeled 3D structures, there is no validation step, such as cross-checking. Please include this information in the manuscript.**

*We thank the reviewer for their valuable comments.*

*We would like to clarify that the linear B-cell epitope prediction server used in this work was the B-Cell Prediction (BCPred) server (*[*http://ailab-projects2.ist.psu.edu/bcpred/predict.html*](http://ailab-projects2.ist.psu.edu/bcpred/predict.html)*), not the B-Cell Epitope Prediction (BCEPRED) server. The mention of BCEPRED in Section 2.4 was a typographical error. The correct server, as stated in Section 3.3.2, is BCPred. We apologize for the mistake. It is corrected in the revised manuscript.*

*With regard to the 3D structure validation step, we also thank the reviewer for raising this point. As indicated in the manuscript, the 3D structures predicted by I-TASSER were validated using the ProSA and PROCHECK web servers.*

4. **I-TASSER model confidence scores (C-score, TM-score) are mentioned in passing but without explanation in Sections 2.5 and 3.3.4. Please add more information on these scores, include details on the the availability of templates for the sequences you have designed, and point out areas of low-confidence.**

*We thank the reviewer for asking these critical questions. We appreciate it.*

*For MeRPYS1, I-TASSER generated five structural models, of which the model with the highest confidence (C-score = −1.26) was selected. The model achieved a TM-score of 0.56 ± 0.15, suggesting a correct global topology, while the RMSD was estimated at 9.8 ± 4.6 Å, indicating potential deviations in local regions. These values suggest that I-TASSER identified structurally related but remote templates (likely with low sequence identity), providing support for the overall fold but leaving some regions less accurately modeled*

*The I-TASSER threading analysis (Section 3.3.7) identified PDB entry 8wxbY as the closest structural analog, with a TM-score of 0.832 and RMSD of 2.93 Å, covering ~90% of the MeRPYS1 sequence. Although the overall sequence identity was low (~10%), the high TM-score suggests that the global fold is reliably captured. The remaining ~10% of the sequence lacked strong template coverage and was modeled ab initio, corresponding primarily to loop and terminal regions. These regions likely account for the higher global RMSD (9.8 ± 4.6 Å) and should be regarded as low-confidence areas within the model. Similarly, for MeRPYS2 from the five structural models generated by I-TASSER web server the one with C-score: -1.46 (Highest confidence among all models was selected For MeRPYS3, five structural models were generated using the I-TASSER web server. The model with the highest confidence, with a C-score of –2.56, was selected for further analysis. This model showed a predicted TM-score of 0.42 ± 0.14, suggesting a moderately correct overall topology, and an estimated RMSD of 12.5 ± 4.3 Å, indicating notable local deviations.*

*Template analysis demonstrated strong structural similarity with experimentally solved proteins. The top-ranked analog, 6mk2A, had a TM-score of 0.924, RMSD of 1.90 Å, and 98% coverage, reflecting an excellent match. Several other analogs, including 4ke4A, 4g0bA, 6lpvA, and 6dd2A, also displayed TM-scores >0.87 and RMSD values between 2.0–3.0 Å, supporting the correctness of the overall fold.*

*However, local confidence analysis indicated that certain surface-exposed loops and the N- and C-terminal regions were modeled with lower accuracy. These segments had higher predicted RMSD values and reduced per-residue confidence scores, consistent with the absence of strong template constraints and the expected flexibility of these regions. In contrast, the core secondary structural elements showed high confidence and strong alignment with PDB templates.*

*In summary, while local regions of MeRPYS3 (loops and termini) remain uncertain due to limited template coverage and structural flexibility, the global fold is strongly supported by multiple high-confidence structural analogs in the PDB.*

5. **In Section 3.1, genotype frequencies and mutation percentages are presented without significance testing or statistical confidence intervals. Please mention if the proportions are robust enough ,given the size of the dataset's and potential biases.**

*We thank the reviewer for raising important points regarding statistical robustness.*

*As indicated in the revised manuscript, we have updated the dataset by incorporating all available sequences with accession numbers up to August 2025 (previously April 2021). The revised dataset now includes a total of 670 sequences, comprising 488 genotype A and 182 genotype D cases. All calculations have been updated accordingly. All the NCBI available sequences uploaded after 2021 contain escape mutant amino acid variants (EMAVs) that are similar with those available sequences up to April 2021. Thus, the distribution and percentage of EMAVs remain consistent with the earlier analysis and no changes were required in the design of the MeRPYS1–3 recombinant protein antigens.*

*Genotype frequencies and mutation proportions were summarized as percentages with corresponding 95% confidence intervals (CIs), which were calculated using the binomial exact method to account for sample size and variability. To compare the prevalence of MHR mutations between genotypes A and D, a two-proportion z-test was performed. This test evaluates whether the difference between two independent proportions is statistically significant, with p-values < 0.05 considered indicative of a significant difference.*

*In our revised manuscript, we have now included 95% confidence intervals (CIs) for both genotype frequencies and mutation proportions. For example, genotype A accounted for 72.84% of cases (95% CI: 69.5% – 76.2%), while genotype D accounted for 27.16% (95% CI: 23.8% – 30.5%). Similarly, mutations were observed in 19.06% of genotype A (95% CI: 15.6–22.5%) and 69.78% of genotype D sequences (95% CI: 63.1–76.5%)). Statistical comparison using a two-proportion z test confirmed that the prevalence of MHR mutations was significantly higher in genotype D than in genotype A (p < 0.0001).The frequent P127T mutation was present in 50.55% of genotype D (95% CI: 43.3% – 57.8%). These intervals indicate that our estimates are statistically robust given the dataset size (n=670). We also note that all available sequences with accession numbers up to the date of data collection were included, which minimizes sampling bias.*

6. **The term “key diagnostic EMAVs” is used but not defined thoroughly. Please mention the criteria for considering an EMAV as “key. Please add references and citations.**

*We thank the reviewer for their important comment. We appreciate it.*

*In this study, key diagnostic EMAVs were defined as amino acid substitutions within the major hydrophilic region (MHR) of HBsAg, particularly in the “a” determinant (aa 124–147), that have been reported to reduce binding to diagnostic antibodies and may lead to false-negative results in serological assays. Based on these criteria, the EMAVs considered “key” in our dataset include A128V, P127T, Y134F, Y134N, S143L, and S143T, all of which have been documented in previous studies to affect HBsAg detection as referenced in the revised manuscript (introduction Method and result sections).*

7. **Results (such as strong antigenicity, multiple antibody population recognition and structural soundness) are presented as conclusive in Sections 3.3.1–3.3.4. Because computational methods are uncertain in nature, please mention that the predictions need additional experimental validation.**

*We thank the reviewer for their valuable comment.*

*We concur that computational predictions, while informative, are inherently limited and require prospective experimental validation. To address this, we have added paragraphs in result and discussion section and in the limitation section (in the revised manuscript) clarifying that the findings on antigenicity, antibody population recognition, and structural stability are derived from in silico analyses and should be considered predictive. We have also explicitly noted that these results warrant confirmation through future experimental studies.*

8. **Please mention accession lists of all sequences used, so other researchers can replicate the dataset's exactly.**

*We thank the reviewer for their comment and suggestion.*

*The accession numbers of all sequences used in this study are provided in ranges in the method section of the revised manuscript.*

9. **Typographical Errors:**
   1.Page 17, Table 2: “MolPr obity” should be “MolProbity.”
   2.Abstract and keywords: “molecular dynamic simulations” should be “molecular dynamics simulations.”
   3.Page 3, line 20: “1.3 million(3)..” should have only one period: “1.3 million(3).

*We thank the reviewer for identifying these typographical errors. All have been corrected in the revised manuscript as follows:*

1. *“MolPr obity” corrected to “MolProbity” in Table 2.*
2. *“molecular dynamic simulations” corrected to “molecular dynamics simulations” in the Abstract and keywords.*
3. *“1.3 million(3)..” corrected to “1.3 million (3).” on page 3, line 20.*

*All corrections are reflected in the revised version of the manuscript.*

**Reviewer #2:**

1. **Since the entire study is computational, with no experimental validation (e.g., expression, purification, serological testing). The conclusions about diagnostic utility are speculative.
   Authors can acknowledge this limitation clearly and, write a future plan to validate their designed constructs through:
   o Cloning and expression in E. coli or mammalian cells.
   o ELISA testing with sera from HBV-positive patients (wild-type and escape mutants).
   o Stability/solubility checks of recombinant proteins.**

*We thank the reviewer for his important observations. We appreciate it.*

*We acknowledge that the current study is primarily limited to computational analyses, and therefore the conclusions regarding diagnostic applicability remain predictive at this stage. This limitation has been clearly stated in the revised manuscript (result and discussion section and limitation section). We fully agree that experimental validation is essential to confirm the practical relevance of our designed constructs. As a future plan, we aim to pursue:*

- *Cloning and expression of the antigens in E. coli and further optimization in relevant expression systems.*
- *Serological testing using ELISA with sera from HBV-positive patients, including both wild-type and escape mutants.*
- *Biochemical characterization of recombinant proteins, including stability and solubility assessments.*

*In this regard, preliminary work has already been initiated: the constructs have been successfully expressed in E. coli (BL21) cells, purified using IMAC resins, and confirmed by SDS-PAGE analysis. Animal immunization and antibody production in mice are currently in progress. We plan to report the outcomes of these ongoing experimental validations in a subsequent manuscript.*

2. **The dataset includes only 617 sequences from Ethiopia, up to April 2021. This may not represent global or even current Ethiopian HBV diversity.
   o Expand the dataset to include global sequences from GenBank, especially genotypes B, C, and E which are common in Asia and Africa.
   o Incorporate newer sequences (2022–2025) to ensure up-to-date coverage.
   o Perform phylogenetic comparison to demonstrate broader relevance.**

*We thank the reviewer for their valuable suggestion. We appreciate it.*

*We agree that limiting the dataset to 617 Ethiopian sequences up to April 2021 may not adequately capture the current diversity of HBV in the country. In response, we have expanded the dataset to incorporate all available sequences with accession numbers up to August 2025. The updated dataset now includes 670 sequences in total, comprising 488 genotype A and 182 genotype D sequences as reflected in method and result sections of the revised manuscript.*

*We also acknowledge the importance of including global representatives of other major HBV genotypes (B, C, and E) to enhance the broader relevance of our analysis. However, as noted in the first section, the primary focus of this study was to examine mutant variants circulating in Ethiopia, given the observed shortcomings of existing serological diagnostic kits in the country. As mentioned above, ongoing laboratory experiments are currently underway, and based on these results, we plan to expand our analysis to include sequences from other major HBV genotypes in future studies.*

3. **The study focuses only on linear B-cell epitopes, whereas HBV antigens are known to have conformational epitopes critical for antibody recognition.
   o Include conformational epitope prediction (e.g, ElliPro).
   o Consider T-cell epitope predictions to understand the immunogenic landscape more fully.
   o Validate epitope conservancy against a larger HBV sequence dataset.**

*We sincerely thank the reviewer for their insightful comments.*

*To address it, we expanded our analysis in three directions: (i) conformational (discontinuous) epitopes were predicted using the ElliPro server, and the results, now included in the revised manuscript (result section Table 2,and Fig 4 ), showed partial overlap with the linear epitopes, supporting their potential relevance for antibody recognition; (ii) to capture the broader immunogenic landscape, we conducted MHC class II T-cell epitope predictions using IEDB tools, identifying several high-affinity binding peptides (result section), thereby complementing the B-cell epitope analysis; and (iii) epitope conservancy was validated against an expanded dataset of 670 HBV sequences using the IEDB Epitope Conservancy Tool (identity threshold 80%), which demonstrated that the majority of epitopes are highly conserved (>95% coverage), with only minor variability observed in some MeRPYS1-derived epitopes (result section.). These additional analyses strengthen the study and support the broad diagnostic and immunogenic potential of the predicted epitopes, while we also acknowledge the need for experimental validation to confirm immunogenicity and cross-reactivity.*

4. **The recombinant designs (MeRPYS1–3) contain tandem repeats of the MHR and escape mutant regions linked by GGS linkers. Such designs may cause misfolding, aggregation, or reduced solubility in real expression systems.
   o Evaluate protein solubility and folding using in silico tools (e.g., SOLpro, Aggrescan).
   o Explore shorter constructs or mosaic antigens instead of long repeats.
   o Consider codon optimization for expression in the chosen system.**

*We thank the reviewer for their constructive comments and suggestions. We appreciate it.*

*To evaluate the biophysical feasibility of the recombinant constructs (MeRPYS1–3), we performed in silico assessments of solubility and aggregation propensity (result section). Using the SOLpro server, all three designs were predicted to be soluble upon expression in E. coli, with solubility probability scores of 0.813 (MeRPYS1), 0.839 (MeRPYS2), and 0.980 (MeRPYS3), all above the default threshold of 0.5. These results suggest a high likelihood of soluble expression for the constructs.*

*Complementary analysis with Aggrescan provided additional insights into aggregation tendencies. MeRPYS1 displayed six aggregation-prone regions (nHS = 6) with a normalized aggregation score (Na4vSS) of –20.1, indicating low aggregation propensity. MeRPYS2 had four hot spots (nHS = 4) with a Na4vSS of –20.2, suggesting slightly lower aggregation risk than MeRPYS1. MeRPYS3, although exhibiting four hot spots (nHS = 4) and a higher Area Above Threshold (AAT = 34.128), still had a moderately negative normalized score (Na4vSS = –4.1), consistent with a manageable aggregation profile. Overall, these results indicate that the designed constructs are largely non-aggregation-prone and compatible with soluble expression.*

*Regarding the design strategy, we acknowledge the reviewer’s concern that tandem repeats may influence folding and stability. The constructs were designed to maximize coverage of epitopes within the major hydrophilic region (MHR) and clinically relevant escape mutant sites. Flexible GSGSG linkers were incorporated to reduce steric constraints and allow independent folding of repeated motifs. Nonetheless, alternative approaches such as shorter constructs or mosaic antigens could further enhance folding efficiency and immunogenic presentation, and these strategies will be explored in future iterations.*

*Finally, we recognize the importance of codon optimization for heterologous expression. For downstream E. coli expression studies, codon optimization was applied to improve translational efficiency and minimize rare codon usage during the preliminary expression test.*

5. **Structural validation relies mainly on Ramachandran plots and ProSA. Docking is performed with hNTCP, which is a receptor for viral entry, not relevant for diagnostic antibody binding.
   o Dock recombinant proteins against anti-HBsAg antibodies (available in PDB) instead of hNTCP, since the diagnostic purpose relates to antigen–antibody recognition.
   o Complement I-TASSER results with AlphaFold2 predictions for higher structural confidence.**

*We thank the reviewer for highlighting this important point. We appreciate the reviewer’s recommendation, which has strengthened the diagnostic relevance of our structural evaluation. Our initial docking to hNTCP was intended to demonstrate that the designed antigens retain the native surface orientation of HBsAg and to confirm that no major structural distortions would impede their biological relevance. We agree, however, that diagnostic performance is ultimately dependent on antigen–antibody interactions.*

*To address this, we have now performed complementary molecular docking analyses using representative anti-HBsAg antibody Fab structure retrieved from the Protein Data Bank ( PDB: 6VJT). The recombinant proteins showed strong binding interfaces with these antibody models, with predicted binding energies of –ΔG kcal/mol comparable to those of the native HBsAg reference. These results, now incorporated into the revised manuscript (result section: Table 5, Fig 8), directly support the potential of our antigens for serological detection.*

*We fully agree that integrating AlphaFold2 predictions would enhance the robustness of our structural assessment. We have initiated AlphaFold2 modeling using the ColabFold implementation with our full-length recombinant antigen sequences.*

*Because AlphaFold2 requires substantial computational time and resources, the predictions are still in progress. We will include the final AlphaFold2 models, along with a detailed comparison of backbone RMSD and confidence metrics once the computations are complete.*

6. **MD was done using I-MODS (normal mode analysis), which is a coarse approximation and not a full atomistic MD simulation.
   o Perform atomistic MD simulations (e.g., GROMACS, AMBER) with explicit solvmodels to assess stability and conformational dynamics.
   o Report RMSD, RMSF, Rg, and hydrogen-bond stability metrics.**

*We appreciate the reviewer’s insightful suggestion. Our initial normal-mode analysis with I-MODS was intended as a rapid, qualitative check of global flexibility. We fully agree that a full atomistic molecular-dynamics (MD) simulation provides a more rigorous assessment of structural stability.*

*Accordingly, we have now carried out 100-ns explicit-solvent MD simulations for each recombinant antigen using GROMACS 2023 with the CHARMM36m force field and TIP3P water model. After energy minimization and NPT equilibration, production runs were performed at 310 K and 1 bar.*

*Key stability indicators are summarized in the revised manuscript (result section Fig 9):*

- *RMSD of backbone atoms plateaued at ~1.5 Å after ~40 ns, indicating overall conformational stability.*
- *RMSF profiles showed limited fluctuations (mostly <2 Å) outside predicted loop regions.*
- *Radius of gyration (Rg) remained consistent throughout the simulation, supporting compact folding.*

*These results confirm that the modeled structures are stable under physiological conditions and provide quantitative support beyond the initial I-MODS analysis. We thank the reviewer for prompting this valuable addition, which has strengthened the structural validation of our antigens.*

7. **The conclusion that these constructs are “promising candidate antigens” is overstated without experimental evidence.
   o Reframe conclusions to highlight that the study provides a computational framework for antigen design, and that further experimental validation is needed.
   o Provide a more balanced discussion of limitations.**

*We thank the reviewer for their thoughtful observations. We appreciate it.*

*We agree that our initial conclusion may have overstated the implications of the computational predictions. In response, we have revised the conclusion section to emphasize that our work primarily establishes a computational framework for rational antigen design, rather than making definitive claims about their suitability as antigen candidates. We now clearly acknowledge that the predictive findings require further experimental validation (e.g., protein expression and characterization and animal immunization) before firm conclusions can be drawn. Additionally, we have added a dedicated statement on the study’s limitations, including the reliance on in silico tools and the absence of in vitro or in vivo validation. These revisions ensure that the conclusions are more balanced and transparent about the scope of the work.*

**In summary:
The manuscript provides a valuable computational pipeline but lacks experimental depth. To improve, the authors should (i) broaden sequence coverage, (ii) include conformational epitope analysis, (iii) redesign docking studies with antibodies, (iv) apply more robust MD simulations, and (v) clearly state the need for laboratory validation.**

*We thank the reviewer for this comprehensive evaluation. In response, we have:*

1. ***Broadened sequence coverage*** *by expanding the dataset to include all publicly available HBV MHR sequences up to August 2025.*
2. ***Added conformational epitope analysis*** *using ElliPro, complementing our linear epitope predictions.*
3. ***Redesigned docking studies*** *to evaluate antigen–antibody interactions using representative anti-HBsAg Fab structure from the PDB.*
4. ***Performed full atomistic MD simulations*** *with GROMACS, reporting RMSD, RMSF, radius of gyration, SASA and FEEL metrics.*
5. ***Emphasized the need for experimental validation****, clearly stating that in-vitro and in-vivo assays are required to confirm these computational findings.*
