# Supplementary material for: In silico design of novel recombinant antigens containing immunologically relevant regions of wild-type and escape mutant variants of HBsAg
Source: PLoS One. 2026 Mar 9;21(3):e0344362. doi: 10.1371/journal.pone.0344362 (PMC12970925; doi:10.1371/journal.pone.0344362)
Supplement: Attachment — Submitted filename: Response_to_Reviewers_auresp_2.docx [file pone.0344362.s007.docx]

**Response to Reviewers**

**Reviewer: General Assessment**
This manuscript presents a computational approach to designing recombinant hepatitis B surface antigens (MeRPYS constructs) that integrate wild-type and immune escape mutant epitopes from circulating Ethiopian HBV strains. The work uses bioinformatics pipelines—epitope mapping, 3D structure modeling, molecular docking, and molecular dynamics simulations—to predict antigen–antibody interactions and structural stability.
The topic is relevant, particularly for regions with high HBV diversity where diagnostic antigen mismatches may occur. The study is methodically organized, and the authors have assembled a comprehensive set of in silico analyses. However, several areas require clarification, validation, and tempering of conclusions. At present, the manuscript remains computationally strong but biologically speculative.

**Response to reviewer’s general assessment**

*We thank the reviewer for their thoughtful and constructive evaluation of our manuscript. We appreciate the reviewer for their positive comments regarding the relevance and organization of the study, as well as the comprehensive nature of the computational analyses presented.*

*We fully agree that computational predictions must be interpreted with appropriate caution. We acknowledge that some statements in the revised submission appeared stronger than the supporting data allowed. In response, we have revised the manuscript to clearly delineate which findings are computational predictions and which require future experimental validation. Additionally, we have expanded the methodological descriptions, clarified the biological rationale behind the design of the MeRPYS constructs, and tempered speculative statements throughout the manuscript.*

*We have also addressed each point raised by the reviewer through additional analyses, clarifications, or text revisions to improve the scientific accuracy and balance of the manuscript.*

**Major comments:**

1. **Lack of Experimental Validation**
   The study’s conclusions about antigenicity and diagnostic potential rely entirely on in silico predictions. While computational studies are valuable, the claims of “diagnostic applicability” or “binding efficiency” cannot be substantiated without in vitro data.
   • The authors should acknowledge more clearly that the findings are hypothesis-generating, not confirmatory.
   • Ideally, expression and immunoreactivity testing of at least one construct (e.g., MeRPYS1) with HBV-positive and negative sera should be included.
   • If laboratory validation is not feasible at this stage, a dedicated future work section should outline a feasible experimental roadmap (expression host, detection method, ELISA validation, etc.).

**Response to Comment 1 (Lack of Experimental Validation)**

*We thank the reviewer for highlighting the relevance of experimental validation. We fully agree that in silico predictions alone cannot confirm the antigenicity, binding efficiency, or diagnostic performance of the constructs.*

- ***Acknowledgment of Hypothesis-Generating Nature***

*We have revised the Abstract, Results, and Discussion sections to explicitly state that the claimed diagnostic applicability of the constructs are* hypothesis-generating *and not confirmatory. All statements related to “diagnostic potential,” “binding efficiency,” or “applicability” have been tempered to reflect the interpretations are based on computational analyses and require experimental validations.*

- ***Experimental Validation***

*We acknowledge the importance of experimental validation of the constructs. At this stage, laboratory experiments such as protein expression, purification, characterization, construct-specific antibody production and immunological assay development have been started. In the meantime, we have revised the manuscript to clearly state these limitations. Our intention is to report the computational findings independently, while the results from experimental validation will be presented in a separate manuscript in the near future.*

- ***Future Experimental Roadmap Added***

*In response to the reviewer’s recommendation, we have added a dedicated “Future Directions” section outlining a feasible validation pathway. This includes:*

- ***Expression of MeRPYS constructs*** *in* E. coli *BL21 (DE3)) using a His-tagged vector system.*
- ***Purification*** *via Ni-NTA affinity chromatography.*
- ***Assessment of antigenicity*** *using indirect ELISA with panels of Ethiopian HBV-positive sera (both wild-type and immune escape variants) and HBV-negative controls.*
- ***Animal immunization and antibody production using Swiss albino mice***
- ***Comparative performance evaluation and validation*** *against marketed HBsAg assays.*

2. **Docking Energy Interpretation**
   The reported docking energies (approximately −1000 kcal/mol) are unrealistically high for antigen–antibody interactions, which typically range between −6 to −15 kcal/mol. This discrepancy likely arises from the scoring scale used by ClusPro or another unnormalized energy unit.
   • The authors should clarify what these values represent, provide normalized binding energies, or use additional scoring such as MM/PBSA to yield physically meaningful results.
   • Without correction, these energy values risk misleading readers about the strength of antigen–antibody binding.

**Response to Comment 2 (Docking Energy Interpretation)**

*We thank the reviewer for their valuable observation. We agree that the reported docking energies (approximately –1000) do not correspond to physical binding energies for antigen–antibody interactions.
We have revised the manuscript to clearly state that the values produced by ClusPro represent* unit-less, weighted scoring functions*, not kcal/mol. These values combine van der Waals, electrostatics, and desolvation terms using algorithm-specific scaling factors. Therefore, they cannot be interpreted as absolute binding free energies. These distinctions are clearly stated in the Methods and Results sections.*

3. **Molecular Dynamics (MD) Simulation Analysis**
   The MD results (RMSD, Rg, SASA, and FEL analyses) are presented, but their biological significance is not sufficiently discussed.
   • Please interpret what structural stabilization implies for antigen–antibody interface robustness and potential folding quality.
   • The discussion should compare fluctuations between the free antigen and the antigen–antibody complex.
   • Indicate simulation length (e.g., 100 ns, 200 ns) and whether it is sufficient for convergence.

**Response to comment 3 (MD Simulation Analysis)**

*We appreciate the reviewer’s insightful comments regarding the interpretation of our MD simulation results. In the revised manuscript, we have expanded the discussion to address these points in detail.*

- ***Biological significance of structural stabilization:***

*We have elaborated on how the observed stabilization in RMSD, Rg, and SASA profiles reflects improved conformational rigidity at the antigen–antibody interface. This stabilization suggests that the complex adopts a more energetically favorable and structurally robust conformation, supporting the likelihood of a stable binding interaction. We further clarified how reduced fluctuations are indicative of proper folding and improved interface integrity.*

- ***Simulation length and convergence:***

*We have specified the total simulation time 110 ns and justify that this duration is sufficient to achieve convergence for the reported parameters. Convergence was confirmed by the plateauing of RMSD values and stable Rg and SASA trends over the final portion of the trajectory.*

4. **Immuno-informatics Prediction Limitations**

The work assumes that predicted epitopes equate to immunogenic or diagnostically relevant epitopes. However, antigenicity predictions do not always correlate with actual antibody recognition due to folding constraints and post-translational modifications.
• The manuscript should temper statements that suggest these epitopes are confirmed immunogenic regions.
• Add references acknowledging prediction limitations (e.g., Jespersen et al., Front. Immunol. 2017).

**Response to comment 4 (Immuno-informatics Prediction Limitations)**

*We thank the reviewer for their important observations. We agree that computationally predicted epitopes do not necessarily correspond to immunogenic or diagnostically relevant regions in experimental settings. Accordingly, we have acknowledged the limitations and revised the manuscript to avoid implying that the predicted epitopes are experimentally confirmed immunogenic sites.*

***Specifically, we have****:*

*• Tempered statements throughout the Results and Discussion to clarify that these epitopes represent predicted rather than validated immunogenic regions.*

*• Added text acknowledging the inherent limitations of epitope prediction tools and the need for subsequent experimental verification.*

*• Cited relevant references highlighting these limitations, including Jespersen et al., Frontiers in Immunology (2017), which discusses the predictive constraints and context-dependent nature of epitope immunogenicity.*

5. **Novelty and Positioning**
   The concept of recombinant HBV antigens combining wild-type and escape mutants is not entirely new. Similar constructs have been reported for diagnostic antigen optimization.
   • The novelty here lies in focusing on Ethiopian HBV genotypes and escape profiles, which should be explicitly stated.
   • Rephrase claims like “first design” to “region-specific computational design of HBsAg diagnostic constructs.”

**Response to comment 5 (Novelty and Positioning)**

*We appreciate the reviewer’s insightful comments. We agree that recombinant HBV antigens combining wild-type and escape mutant sequences have been described previously in the context of diagnostic antigen optimization. Specifically, the intention of our work was to incorporate Ethiopian HBV genotype distribution and region-specific escape mutation profiles, which, to our knowledge, have not been systematically integrated into diagnostic antigen design and engineering.*

*We have revised the manuscript to clearly state that the novelty of our study lies in the region-specific computational design of HBsAg diagnostic constructs.*

6. **Genotype Analysis and Sequence Statistics**
   The frequency comparison between genotypes A and D is mentioned but lacks details on statistical testing or visualization.
   • Include the number of sequences analyzed per genotype, the source database, and statistical significance (e.g., chi-square test).
   • A simple heatmap or frequency bar chart summarizing amino acid substitutions would improve clarity.

**Response to Comment 6 (Genotype Analysis and Sequence Statistics***)*

*We thank the reviewer for emphasizing the need for additional detail regarding the genotype frequency comparison. We would like to clarify that in the previous manuscript, the number of sequences analyzed per genotype and the source database (NCBI) were specified in the Results and Methods sections, respectively. The statistical significance of the comparisons, assessed using a two-proportion z-test, was reported in both the Methods and Results sections. A frequency bar chart was incorporated in the Results section, illustrating the top ten most prevalent mutant amino acid variants in the “a” determinant region of HBsAg for genotypes A and D in Ethiopia.*

7. **Expression Feasibility**

- Although the design incorporates linkers and tags, expression feasibility is not evaluated.
- Add codon optimization results (CAI, GC%) for a likely host (e.g., E. coli).
- Predict solubility and signal peptide presence using tools such as SOLpro or SignalP.

**Response to comment 7 (Expression Feasibility)**

*We appreciate the reviewer’s insightful comment emphasizing the need to evaluate the expression feasibility of the designed constructs. We have now incorporated comprehensive analyses addressing codon usage (CAI and GC content), and signal peptide assessment. However, the solubility prediction result was left as it was included during the previous revision.*

**Minor comments:**

1. **Clarity of Methods:**

- Specify the exact versions and parameters of each tool used (IEDB, VaxiJen, ClusPro, GROMACS, etc.).
- Define the basis for selecting epitopes—thresholds, prediction cutoffs, or ranking criteria.

**Response**:

*We thank the reviewer for their important comments regarding methodological clarity. In the revised manuscript, we have now specified the exact versions of all computational tools and servers used. Specifically, the updated IEDB tools (2024 release) were used for epitope prediction, ClusPro 2.0 was employed for molecular docking, and GROMACS 2024 was utilized for the molecular dynamics simulations. All thresholds, prediction cutoffs, or ranking criteria for epitope selection were provided in the revised Methods section.*

2. **Figures:**
   • Figures 5–8 (structural modeling and docking) can be combined into a single composite workflow figure.

- Figure legends are verbose and sometimes repeat information; make them more concise.
- Include residue labels for key contact sites in docking visuals.

**Response**

*We appreciate the reviewer’s thoughtful suggestion to merge Figures 5–9 into a single composite workflow figure. While this approach is conceptually appealing, it is not feasible in our case because the study presents data for three different recombinant proteins, resulting in more than 20 individual figures. Combining all of these into one workflow figure would compromise clarity and readability.*

*However, we have incorporated residue labels for key contact sites in Fig 9 as recommended. Additionally, the figure legends have been revised to be more concise and easier for the readers to follow.*

3. **Table Presentation:**

Some tables lack clear titles and units. Include binding energy units and consistent decimal formatting.

**Response:**

*We appreciate the reviewer’s detailed observations regarding table clarity. In the revised manuscript, we have thoroughly updated all relevant tables to improve readability and consistency. Specifically, descriptive titles have been added to each table to clearly indicate their content and purpose. Decimal formatting and unit usage have been standardized across all tables to ensure uniform presentation of numerical values.*

4. **Statistical Reporting:**

- Provide mean ± SD for numerical data where applicable.
- Report how many simulation replicates were performed.

**Response:**

*We thank the reviewer for their comment. We would like to clarify that the manuscript does not include any datasets that require presentation as mean ± SD. Additionally, the molecular dynamics simulations were performed once for each antigen-antibody complex, following standard practice for exploratory structural analyses. We have added a clarification in the Methods section to avoid any misunderstanding.*

5. **References:**

- Check recent studies on HBV escape mutations and diagnostic antigen design (2023–2024) to strengthen the discussion.
- Ensure all URLs or database accessions are up to date and functional.

**Responses:**

*We thank the reviewer for their important suggestions. We have now conducted a thorough literature review for studies published in 2023–2024 on HBV immune-escape mutations and diagnostic antigen design. Recent papers have been added to the discussion section — highlighting how their findings on escape-mutant epitopes and antigen reactivity inform and support our design strategy. Additionally, we have systematically checked every URL and database accession in the manuscript. All links are now verified as functional, and database entries are updated to their current versions. These updates are reflected in the revised manuscript.*

**Editorial and Language Comments:**

The manuscript is generally understandable but requires polishing for grammar and flow. Common issues include missing articles, verb–subject disagreements, and repetition.

Example:
“The small hepatitis B surface antigen (HBsAg), which consists of major hydrophilic region and ‘a’ determinant region…”

Suggested Correction:

“The small hepatitis B surface antigen (HBsAg) comprises the major hydrophilic region (MHR) and the ‘a’ determinant.”

Example:
“This study is the first study to design…”

Suggested Correction:

“This study is the first to design…”

Example:
“Each blue dot represents a single amino acid residue.”

Suggested Correction:

“Each blue dot corresponds to an amino acid residue.”

Example:
“These results confirmed that the designed antigens are immunologically reactive.”

Suggested Correction:

“These results suggest that the designed antigens are potentially immunoreactive.”

Example:
“Proteins were constructed by assembling ten tandem copies of MHR segments.”

Suggested Correction:

“Proteins were constructed from ten tandem repeats of MHR segments.”

Example:
“Physiochemical”
Suggested Correction:

“Physicochemical”
Example:
“A flexible linker was added to connect two epitopes”

Suggested Correction:

“Flexible linkers (GSGSG) were introduced to maintain structural flexibility between epitopes.”

**Response to Editorial and Language Comments**

*We thank the reviewer for their insightful comments regarding grammar, clarity, and overall flow of the manuscript. We carefully revised the entire text to address missing articles, verb–subject disagreements, and repetitive or awkward phrasing. All suggested corrections provided by the reviewer have been incorporated, and similar issues throughout the manuscript were systematically edited for consistency and readability.*

*Specifically, we have:*

- *Revised descriptions of HBsAg regions for clarity (e.g., “The small hepatitis B surface antigen (HBsAg) comprises the major hydrophilic region (MHR) and the ‘a’ determinant”).*
- *Improved figure descriptions for precision (e.g., “Each blue dot corresponds to an amino acid residue”).*
- *Adjusted interpretative statements to avoid over claiming (e.g., “These findings suggest that the designed antigens potentially possess strong antigenic properties”).*
- *Clarified protein design methodology (e.g., “Proteins were constructed from ten tandem repeats of “a” determinant regions”).*
- *Refined methodological explanations (e.g., “Flexible linkers (GSGSG) were introduced to maintain structural flexibility between epitopes”).*

**General stylistic suggestions:**

- Maintain consistent use of either American or British English.
- Avoid redundancy (“HBsAg antigen” → “HBsAg”).
- Simplify long sentences—many exceed 35 words and can be split for readability.
- Ensure that all acronyms are defined upon first use.

**Response to general stylistic suggestions**

*We thank the reviewer for their valuable stylistic recommendations. We carefully revised the manuscript to enhance consistency, clarity, and readability.*

*Specifically, we have:*

- *Standardized English usage by adopting American English throughout the manuscript to ensure uniformity.*
- *Eliminated redundant phrasing, including instances such as “HBsAg antigen,” which have been corrected to “HBsAg.”*
- *Shortened long sentences, especially those exceeding 30–35 words, by restructuring them into clearer and more concise statements.*
- *Defined all acronyms upon their first usage and ensured their consistent use throughout the manuscript.*

**Decision: Major Revision**

- While the computational analyses are detailed and relevant, the manuscript requires:
- Clarified energy interpretation and docking validation,
- Deeper discussion of MD results,
- Explicit acknowledgment of the limitations of predictive modeling,
- Clearer statistical and methodological descriptions, and
- Careful language editing.

Experimental data are not mandatory for PLOS ONE if the modeling is robust, but the conclusions must be scaled down to reflect a computational hypothesis rather than proven diagnostic efficacy.

**Response to Reviewer’s Major Revision Requirements**

*We thank the reviewers for their thorough evaluation of our manuscript and for outlining the key areas requiring improvement. We have carefully addressed each point to strengthen the scientific rigor, clarity, and interpretability of the study.*

*Specifically, we have made the following major revisions:*

- *Clarified docking energy interpretation and validation:*

*We revised the docking section to explain the scoring functions more accurately, clarified that the reported energies are in arbitrary units. We also tempered interpretations to avoid overstating binding strength.*

- *Provided deeper discussion of MD simulation results:*

*The MD analysis has been expanded to include more detailed interpretation of RMSD, RMSF, Rg, SASA, and FEL profiles, with more clarified links to antigen stability, conformational behavior, and antibody interaction potential.*

- *Added explicit acknowledgment of predictive modeling limitations:*

*A dedicated subsection now discusses the inherent limitations of computational modeling, including force-field assumptions, simulation timescales, and the absence of experimental validation. We emphasize that the findings represent predictive insights rather than confirmed biological outcomes.*

- *Conducted comprehensive language editing:*

*The entire manuscript has been thoroughly polished to improve clarity, grammar, and flow. Stylistic inconsistencies and repetitive phrasing have also been addressed.*

*Finally, we fully acknowledge that experimental confirmation is not required for PLOS ONE, but, as advised, we have appropriately scaled down the conclusions to reflect a computational framework that supports a hypothesis rather than demonstrating diagnostic efficacy.*
