# Supplementary material for: In silico design of novel recombinant antigens containing immunologically relevant regions of wild-type and escape mutant variants of HBsAg
Source: PLoS One. 2026 Mar 9;21(3):e0344362. doi: 10.1371/journal.pone.0344362 (PMC12970925; doi:10.1371/journal.pone.0344362)
Supplement: Attachment — Submitted filename: PONE-D-25-38657_R2_SB_12.25.25.docx [file pone.0344362.s006.docx]

**Overall Assessment**

This is a well-structured, comprehensive in silico study addressing an important and regionally relevant problem: the impact of HBsAg escape mutant variants on HBV diagnosis in Ethiopia. The computational workflow is extensive, logically organized, and technically sound, covering sequence analysis, antigen design, epitope prediction, structure modeling, docking, and MD simulations.

The manuscript demonstrates strong effort, depth, and clarity of intent, and it is suitable for a journal like PLOS ONE. However, several methodological, interpretational, and presentation-related issues need to be addressed before acceptance.

**Recommendation**

**Minor Revision**

This recommendation is due to the following reasons:

- Some methodological choices need stronger justification
- There is over-interpretation of in silico results
- Several sections require language polishing and structural tightening
- Some figures/tables and claims need clarification or correction

With proper revisions, the manuscript could become publishable.

**Section-by-Section Revision Suggestions**

**1. Abstract**

**Issues:**

- Some statements slightly overstate conclusions from purely computational work.
- The abstract is long and could be tightened.

**Suggestions:**

- Replace phrases like:
  - “demonstrated the probable antigen-antibody interactions”

with

- “suggested potential antigen-antibody interactions”
- Consider shortening background statistics to focus more on novelty and results.

**2. Introduction**

**Strengths:**

- Strong epidemiological context
- Clear rationale for region-specific antigen design

**Issues:**

- Some repetition regarding diagnostic escape mutants
- Overuse of global HBV statistics where regional context would be stronger

**Suggestions:**

- Reduce redundancy in paragraphs discussing EMAVs
- Clearly state what gap this study fills compared to previous in silico HBV antigen studies
- Explicitly clarify why polyclonal antibody production is the chosen downstream goal (diagnostics vs vaccines)

**3. Methods**

**Key concerns:**

**a) Sequence dataset justification**

- Exclusion of genotype E (KT367574) needs stronger justification, as even rare genotypes may influence diagnostic design.
- Clarify whether the dataset represents unique patients or multiple sequences per patient.

**b) Statistical analysis**

- Two-proportion z-test is mentioned but:
  - No correction for multiple testing
  - No effect size discussion

**c) Epitope prediction thresholds**

- Choice of:
  - BCPRED cutoff
  - ELIPRO score ≥ 0.5
  - MHC-II percentile ≤ 0.1

should be justified with citations.

**d) Molecular dynamics**

- Only one MD run per complex was performed.
  - This is a limitation and should be acknowledged more explicitly.

**Suggestions:**

- Add a limitations paragraph within Methods or Discussion explaining:
  - Single-run MD simulations
  - Reliance on murine MHC-II only
- Clarify reproducibility (random seeds, repeatability)

**4. Results and Discussion**

This is the strongest section scientifically, but also where over-interpretation occurs.

**a) EMAV prevalence analysis**

- Very solid
- Consider adding a supplementary table listing all EMAVs and frequencies

**b) Epitope prediction**

- Statements like:

“potentially induce polyclonal antibody production”

should be softened to

“may support polyclonal antibody responses”

- Clarify that epitope duplication ≠ guaranteed immunodominance

**c) Structural modeling**

- Well done
- However, Ramachandran plot interpretation should avoid phrases like “well-formed” and instead use “acceptable stereochemical quality”

**d) Docking and MD simulations**

**Issues:**

- Docking scores are relative, but sometimes discussed as indicators of binding strength
- RMSD values reported (<1.75 Å) seem unusually low for large antigen–antibody complexes—needs clarification:
  - backbone only?
  - antigen only?
- Free-energy landscape units described as “arbitrary units” without explanation

**Suggestions:**

- Explicitly state:
  - RMSD atom selection
  - Docking is qualitative, not quantitative
- Tone down claims of “strong binding” and use “stable interaction patterns”

**5. Conclusion**

**Issues:**

- Slight repetition of Results
- Some statements verge on confirmatory tone

**Suggestions:**

- Focus on what was achieved computationally
- End with a stronger emphasis on hypothesis generation

**6. Limitations and Future Directions**

**Strength:**

- Honest and transparent

**Suggestions:**

- Add:
  - Limitation of murine MHC predictions for human diagnostics
  - Lack of glycosylation modeling (important for HBsAg)

**7. Language and Style**

**Common issues:**

- Article misuse (“the”, “a”)
- Verb tense inconsistency
- Occasional long, dense sentences

**Recommendation:**

- Professional English language editing strongly recommended
- Especially for Results and Discussion

**Final Decision**

Rationale:

- The science is solid and meaningful
- The computational workflow is impressive
- However, claims must be strengthened, and methodological clarity improved
- Language polishing is necessary for journal standards
