# Supplementary material for: In silico design of novel recombinant antigens containing immunologically relevant regions of wild-type and escape mutant variants of HBsAg
Source: PLoS One. 2026 Mar 9;21(3):e0344362. doi: 10.1371/journal.pone.0344362 (PMC12970925; doi:10.1371/journal.pone.0344362)
Supplement: Attachment — Submitted filename: Response_to_Reviewers_auresp_3.docx [file pone.0344362.s009.docx]

**Response to Reviewers**

**Reviewer’s Overall Assessment:**

This is a well-structured, comprehensive in silico study addressing an important and regionally relevant problem: the impact of HBsAg escape mutant variants on HBV diagnosis in Ethiopia. The computational workflow is extensive, logically organized, and technically sound, covering sequence analysis, antigen design, epitope prediction, structure modeling, docking, and MD simulations.

The manuscript demonstrates strong effort, depth, and clarity of intent, and it is suitable for a journal like PLOS ONE. However, several methodological, interpretational, and presentation-related issues need to be addressed before acceptance.

**Response to Reviewer’s Overall Assessment:**

*We thank the reviewer for the positive and thoughtful overall assessment of our manuscript. We appreciate the recognition of the study’s structure, computational depth, and regional relevance, as well as the evaluation of the in silico workflow as technically sound and logically organized.*

*We acknowledge the reviewer’s comments regarding the need for improvements in methodological clarity, interpretation, and presentation. Accordingly, we have carefully revised the manuscript to address these issues, including clearer methodological descriptions, refined interpretation of computational findings, appropriate scaling of conclusions, and language editing. We believe these revisions have strengthened the manuscript and improved its suitability for publication in PLOS ONE.*

**Recommendation**

**Minor Revision**

This recommendation is due to the following reasons:

- Some methodological choices need stronger justification
- There is over-interpretation of in silico results
- Several sections require language polishing and structural tightening
- Some figures/tables and claims need clarification or correction

With proper revisions, the manuscript could become publishable.

**Section-by-Section Revision Suggestions**

**1. Abstract**

**Issues:**

- Some statements slightly overstate conclusions from purely computational work.
- The abstract is long and could be tightened.

**Suggestions:**

- Replace phrases like:
  - “demonstrated the probable antigen-antibody interactions”

with

- “suggested potential antigen-antibody interactions”
- Consider shortening background statistics to focus more on novelty and results.

**Response:**

*We thank the reviewer for their constructive suggestions. We have revised the abstract to adopt a more cautious tone, replacing phrases such as “demonstrated the probable antigen-antibody interactions” with “suggested potential antigen-antibody interactions” to accurately reflect the computational nature of the study. Additionally, the abstract has been shortened by condensing background statistics and emphasizing the novelty, design, and key results of the study.*

**2. Introduction**

**Strengths:**

- Strong epidemiological context
- Clear rationale for region-specific antigen design

**Issues:**

- Some repetition regarding diagnostic escape mutants
- Overuse of global HBV statistics where regional context would be stronger

**Suggestions:**

- Reduce redundancy in paragraphs discussing EMAVs
- Clearly state what gap this study fills compared to previous in silico HBV antigen studies
- Explicitly clarify why polyclonal antibody production is the chosen downstream goal (diagnostics vs vaccines)

**Response:**

*We thank the reviewer for recognizing the strengths, including the clear epidemiological context and rationale for region-specific antigen design. We revised the section to reduce redundancy in the discussion of diagnostic escape mutants and to focus more on the regional context of HBV variants in Ethiopia. We also clarified the specific gap addressed by this study in comparison to previous in silico HBV antigen research. Furthermore, we tried to explicitly justify the focus on polyclonal antibody production as the downstream goal, emphasizing its relevance for the development of region-specific diagnostic assays.*

**3. Methods**

**Key concerns:**

**a) Sequence dataset justification**

- Exclusion of genotype E (KT367574) needs stronger justification, as even rare genotypes may influence diagnostic design.
- Clarify whether the dataset represents unique patients or multiple sequences per patient.

**Response:**

*We thank the reviewer for these important comments.*

- ***Exclusion of genotype E (KT367574****): This sequence was excluded as it represents a single entry, limiting its applicability for reliable antigen design. While we recognize that rare genotypes may impact diagnostic considerations, the study focuses on the predominant HBV variants circulating in Ethiopia to ensure region-specific relevance and robust computational predictions. This rationale has been clarified in the Methods section.*
- ***Uniqueness of the dataset:*** *All 682 HBsAg sequences included in the analysis represent unique patient samples. We have updated the manuscript to explicitly state this to avoid ambiguity regarding sequence redundancy.*

**b) Statistical analysis**

- Two-proportion z-test is mentioned but:
  - No correction for multiple testing
  - No effect size discussion Response:

**Response:**

*We thank the reviewer for raising important statistical considerations. The two-proportion z-test was used to evaluate differences in prevalence between groups for a limited number of predefined, hypothesis-driven comparisons; therefore, the analysis was not exploratory in nature and no formal multiple-testing correction was initially applied. To improve clarity and transparency, we have now added a detailed description of the statistical analysis workflow under a dedicated subsection in the Methods section.*

*Regarding effect size, we agree that statistical significance alone does not adequately reflect the magnitude of the observed differences. Accordingly, we have included effect size estimates by reporting the absolute difference in proportions (Δp). We believe that the correction provides a clearer interpretation of the practical relevance of the findings beyond p-values alone.*

*These revisions have been incorporated into the Methods and Results sections to strengthen the statistical rigor and transparency of the study*.

**c) Epitope prediction thresholds**

- Choice of:
  - BCPRED cutoff
  - ELIPRO score ≥ 0.5
  - MHC-II percentile ≤ 0.1

should be justified with citations.

**Response:**

*We thank the reviewer for their suggestions regarding justification of prediction thresholds. We added appropriate references in the Methods to support the choice of cutoffs for each prediction tool.*

- ***BCPred cutoff:***

*For linear B-cell epitope prediction, high scoring thresholds are frequently applied to enrich for likely antigenic regions and improve specificity in epitope selection. Similar immune-informatics studies have selected predicted linear epitopes from BCPred with scores above stringent thresholds (e.g., ≥0.9) to focus on high-confidence candidates, consistent with practices in subunit vaccine design pipelines.*

- ***ElliPro score ≥ 0.5:***

*ElliPro computes a Protrusion Index (PI) score for each predicted epitope based on structural protrusion, where higher PI values indicate greater likelihood of solvent exposure and antibody accessibility. The tool’s default scoring framework and prior applications adopt a minimum PI cutoff of ≥0.5 to retain structurally accessible conformational epitopes.*

- ***MHC-II percentile ≤ 0.1:***

*Selection of high-affinity MHC class II binders based on stringent percentile rank thresholds is a common and recommended practice in immuno-informatics. Although the IEDB analysis resource generally suggests a percentile rank ≤10% for inclusive screening, more stringent percentile rank cutoffs (e.g., ≤0.5 or ≤1%) are widely used in recent vaccine design studies to prioritize the top predicted binders for experimental follow-up. Percentile rank filtering thereby enhances specificity across diverse alleles.*

*We have incorporated these citations into the revised Methods section to justify our analytic choices.*

**d) Molecular dynamics**

- Only one MD run per complex was performed.
  - This is a limitation and should be acknowledged more explicitly.

**Suggestions:**

- Add a limitations paragraph within Methods or Discussion explaining:
  - Single-run MD simulations
  - Reliance on murine MHC-II only
- Clarify reproducibility (random seeds, repeatability)

**Response:**

*We thank the reviewer for their important comments and suggestions. We acknowledge that performing a single MD prediction run per complex is a limitation of this study. Accordingly, we tried to explicitly state this in the limitation section.*

*We also added a limitation clarifying that T-cell epitope modeling was restricted to murine MHC class II alleles.*

*Regarding reproducibility, we clarified in the Methods that all MD simulations were conducted using fixed input structures and predefined parameters, with stochastic components generated using default random seeds, ensuring that the simulation workflow is fully repeatable.*

**4. Results and Discussion**

This is the strongest section scientifically, but also where over-interpretation occurs.

**a) EMAV prevalence analysis**

- Very solid
- Consider adding a supplementary table listing all EMAVs and frequencies

**Response:**

*We thank the reviewer for the positive assessment of the Results and Discussion section, particularly the EMAV prevalence analysis. We added a more detailed description of the calculation procedures and interpretative framework used to assess EMAV prevalence. We agree that careful interpretation is essential to avoid overstatement; accordingly, we have revised the manuscript to further moderate the language and ensure that all conclusions are strictly supported by the data. We also included a new supplementary table listing all identified EMAVs and their corresponding frequencies to enhance transparency and facilitate independent evaluation of the findings.*

**b) Epitope prediction**

- Statements like:

“potentially induce polyclonal antibody production”

should be softened to

“may support polyclonal antibody responses”

- Clarify that epitope duplication ≠ guaranteed immunodominance

**Response:**

*We thank the reviewer for this important comment regarding the interpretation of the epitope prediction results. We revised the wording throughout the manuscript to adopt more cautious and appropriate language. Specifically, statements such as “potentially induce polyclonal antibody production” have been softened to “may support polyclonal antibody responses” to better reflect the predictive and theoretical nature of the analysis. In addition, we have clarified that epitope duplication alone does not guarantee immune-dominance..*

**c) Structural modeling**

- Well done
- However, Ramachandran plot interpretation should avoid phrases like “well-formed” and instead use “acceptable stereochemical quality”

**Response:**

*We thank the reviewer for their positive feedback and suggested corrections. Accordingly, we incorporated the suggested corrections in the revised manuscript*

**d) Docking and MD simulations**

**Issues:**

- Docking scores are relative, but sometimes discussed as indicators of binding strength
- RMSD values reported (<1.75 Å) seem unusually low for large antigen–antibody complexes—needs clarification:
  - backbone only?
  - antigen only?
- Free-energy landscape units described as “arbitrary units” without explanation

**Suggestions:**

- Explicitly state:
  - RMSD atom selection
  - Docking is qualitative, not quantitative
- Tone down claims of “strong binding” and use “stable interaction patterns”

**Response:**

*We thank the reviewer for their important and constructive comments regarding the interpretation of the docking and MD simulation results. We have revised the manuscript to improve clarity, methodological transparency, and to ensure appropriate interpretation of the computational findings.*

*First, we acknowledge that docking scores are inherently relative and do not represent absolute binding affinities. Accordingly, we have revised the text to explicitly state that molecular docking energies were used as relative binding energies to compare relative binding poses and interaction patterns rather than to quantify binding strength. All statements implying “strong binding” have been toned down and replaced with more appropriate terms such as “stable interaction patterns.*

*Second, regarding the reported RMSD values (<1.75 Å), we have clarified the atom selection used for RMSD calculations. Specifically, RMSD values were computed using backbone atoms (Cα). This clarification has been added to the Methods and Result sections to avoid potential misinterpretation, as RMSD values for large multi-domain complexes are expected to be higher when all atoms are included.*

*Third, we have clarified the interpretation of the free-energy landscape (FEL) analysis. The term “arbitrary units” refers to relative free-energy values derived from probability density distributions of conformational states and is intended for comparative visualization of conformational stability, rather than absolute energetic quantification. This explanation has now been explicitly stated in the revised manuscript.*

**5. Conclusion**

**Issues:**

- Slight repetition of Results
- Some statements verge on confirmatory tone

**Suggestions:**

- Focus on what was achieved computationally
- End with a stronger emphasis on hypothesis generation

**Response:**

*We thank the reviewer for this valuable feedback. In response, we have revised the Conclusion section to reduce repetition of the Results and to avoid a confirmatory tone. The revised Conclusion now focuses on summarizing what was achieved through the computational analyses, rather than reiterating specific numerical outcomes.*

*The revised text now clearly frames the findings as hypothesis-generating, highlighting that the computational results provide a rationale for subsequent experimental validation, including protein expression, immunogenicity assessment, and diagnostic assay development.*

**6. Limitations and Future Directions**

**Strength:**

- Honest and transparent

**Suggestions:**

- Add:
  - Limitation of murine MHC predictions for human diagnostics
  - Lack of glycosylation modeling (important for HBsAg)

**Response**:

*We thank the reviewer for recognizing the transparency of the Limitations and Future Directions section. In response to the suggestions, we have expanded this section to explicitly acknowledge additional methodological constraints.*

*First, we now clearly state that T-cell epitope predictions were limited to murine MHC class II alleles. While this approach is appropriate for preliminary immunogenicity assessment and planned animal experiments, we acknowledge that extrapolation to human immune responses is limited and will require validation using human HLA alleles in future studies.*

*Because the recombinant antigens are intended for expression in a bacterial expression system, where glycosylation does not occur, glycosylation prediction was not incorporated into the current computational pipeline.*

**7. Language and Style**

**Common issues:**

- Article misuse (“the”, “a”)
- Verb tense inconsistency
- Occasional long, dense sentences

**Recommendation:**

- Professional English language editing strongly recommended
- Especially for Results and Discussion

**Response:**

*We thank the reviewer for highlighting the language and stylistic issues in the manuscript. In response, the manuscript has undergone thorough revision to address article usage (e.g., “the,” “a”), verb tense consistency, and sentence clarity. Long and dense sentences, particularly in the Results and Discussion sections, have been restructured to improve readability and precision.*

*In addition, the manuscript has been carefully edited to ensure professional scientific English throughout, with particular attention to clarity and consistency in the Results and Discussion sections. These revisions were made to enhance the overall quality, coherence, and accessibility of the manuscript.*

**Final Decision**

Rationale:

- The science is solid and meaningful
- The computational workflow is impressive
- However, claims must be strengthened, and methodological clarity improved
- Language polishing is necessary for journal standards

**Response:**

*We sincerely thank the editor and reviewers for their careful evaluation of our manuscript and for recognizing the scientific relevance of the study and the strength of the computational workflow. We appreciate the constructive feedback provided.*

*In response, we have strengthened the manuscript by improving methodological clarity, particularly in the description and interpretation of docking and molecular dynamics analyses, and by carefully scaling claims to reflect the predictive and hypothesis-generating nature of the computational findings. All conclusions have been revised to avoid over interpretation and to align with the scope of in silico analyses.*

*Additionally, the manuscript has undergone comprehensive language polishing to meet the journal’s standards, with focused editing to improve clarity, consistency, and readability throughout the text.*

*We believe these revisions have substantially improved the quality and rigor of the manuscript and have addressed all concerns raised in the final decision*
