# Supplementary material for: In silico design of novel recombinant antigens containing immunologically relevant regions of wild-type and escape mutant variants of HBsAg
Source: PLoS One. 2026 Mar 9;21(3):e0344362. doi: 10.1371/journal.pone.0344362 (PMC12970925; doi:10.1371/journal.pone.0344362)
Supplement: Attachment — Submitted filename: Response to Reviewer Comments.docx [file pone.0344362.s010.docx]

**Response to Reviewers Comment**

Dear Authors,

This manuscript presents a comprehensive *in silico* design and characterization of three recombinant HBsAg-derived antigens (MeRPYS1–3) intended for improved HBV diagnostic applications in Ethiopia. The study integrates sequence epidemiology, epitope prediction, structural modeling, molecular docking, and molecular dynamics simulations.

The work addresses a relevant public-health and diagnostic problem, particularly in regions with high HBV burden and genotype diversity. The computational pipeline is extensive and technically competent. However, the manuscript currently over interprets *in silico* findings, lacks clarity in several methodological justifications, and requires significant tightening of claims to meet PLOS ONE’s standards for methodological rigor and reproducibility.

**Response to general comments**

*We sincerely thank the reviewer for their careful evaluation of our work and for their constructive and insightful comments. We appreciate their recognition regarding the relevance of this work to hepatitis B virus (HBV) diagnostics in high-burden settings such as Ethiopia. We also appreciate their acknowledgment of the breadth and technical competence of the computational pipeline employed.*

*In response to their concerns, we have substantially revised the manuscript to improve clarity, rigor, and reproducibility. Specifically, we have carefully reviewed all interpretations derived from in silico analyses and have moderated statements that could be construed as over interpretation. Claims regarding diagnostic performance and biological relevance are now explicitly framed as computational predictions that require experimental validation, and speculative language has been removed or appropriately qualified throughout the text.*

*We have also expanded and clarified the methodological justifications at each stage of the computational workflow. The rationale for sequence selection, epitope prediction strategies, structural modeling approaches, docking protocols, and molecular dynamics simulation parameters is now described in greater detail, with additional references provided where appropriate. These revisions were made to ensure transparency, reproducibility, and alignment with PLOS ONE’s methodological standards.*

*Furthermore, the manuscript has been carefully edited to improve coherence and conciseness. Redundant descriptions were removed, and sections were reorganized to better distinguish between methodological procedures, results, and interpretation. We believe these changes significantly strengthen the manuscript and more accurately reflect the scope and limitations of in silico investigations.*

*We are grateful for the reviewer’s thoughtful feedback, which enabled us improve the quality and scientific rigor of this work. We hope that the revised manuscript now satisfactorily addresses the reviewer’s concerns and meets the standards required for publication in PLOS ONE.*

**Comments (Should be addressed)**

**1. Over-interpretation of *in silico* predictions**

While the authors repeatedly acknowledge the lack of experimental validation, several conclusions still go beyond what is justified by computational analyses alone.

**Examples:**

- Statements suggesting “stable antigen–antibody interactions” and “supporting candidacy for polyclonal antibody production” are not experimentally substantiated.
- MD simulation stability is interpreted as evidence of functional binding, which is not equivalent to biochemical affinity or immunoreactivity.

**Recommendation:** Rephrase conclusions throughout the Results, Discussion, and Abstract to emphasize hypothesis-generating rather than confirmatory outcomes. Avoid language implying functional efficacy.

**Response to Comment 1: Over-interpretation of *in silico* predictions**

*We thank the reviewer for their important and well-taken comments. We agree that, in the original version of the manuscript, certain statements extended beyond what can be justified by computational analyses alone.*

*In response, we have systematically revised the Abstract, Results, and Discussion sections to ensure that all conclusions are framed as hypothesis-generating rather than confirmatory. Language implying experimentally validated stability, functional antigen–antibody binding, or diagnostic efficacy has been removed or appropriately qualified.*

*Specifically, statements suggesting “stable antigen–antibody interactions” and “supporting candidacy for polyclonal antibody production” have been rephrased to emphasize that these observations represent computationally predicted interaction patterns and theoretical stability under simulated conditions, rather than experimentally substantiated biochemical affinity or immune-reactivity.*

*We have also clarified the interpretation of molecular dynamics (MD) simulations throughout the manuscript. MD-derived stability metrics are now explicitly described as reflecting the behavior of modeled complexes within a computational framework, and not as evidence of functional binding or immune recognition in biological systems. Any implication that MD stability equates to functional efficacy has been removed.*

*Finally, the Abstract and Conclusions have been revised to clearly state that the in silico analyses are intended to generate testable hypotheses and inform experimental prioritization, with empirical validation required to assess antigenicity and diagnostic utility.*

*We believe these revisions adequately address the reviewer’s concerns and align the manuscript with PLOS ONE’s expectations regarding appropriate interpretation of computational findings.*

**2. Limited novelty relative to existing *in silico* multi-epitope antigen studies**

The manuscript claims to be the first to computationally design region-specific HBV antigens. However:

- Similar multi-epitope and mutation-inclusive HBsAg designs have been reported previously (including outside Ethiopia).
- The novelty here appears to be regional sequence prioritization, not the computational strategy itself.

**Recommendation:** Clarify novelty explicitly:

- Emphasize Ethiopia-specific EMAV prevalence data
- Avoid blanket “first study” claims unless clearly justified with citations

**Response to Comment 2: Clarification of Novelty**

*We thank the reviewer for their comments and have revised the manuscript accordingly. We have removed statements claiming this work as the “first” computational design of HBV antigens. The novelty of our study lies in the integration of Ethiopia-specific HBsAg EMAV prevalence data into antigen design, enabling prioritization of variants most relevant for the local population. The computational approach itself is established.*

**3. Statistical analysis is correct but conceptually underused**

The genotype A vs D comparison is statistically strong (z = 12.44, Δp = 50.7%), but:

- This result is not integrated mechanistically into antigen design decisions.
- It is unclear how genotype-specific mutation prevalence quantitatively informed MeRPYS1 vs MeRPYS2 composition.

**Recommendation:** Explicitly link statistical findings to design rationale:

- Why do MeRPYS1 and MeRPYS2 differ?
- How does genotype D’s higher EMAV burden influence antigen architecture?

**Response to Comment 3: integration of Statistical analysis result with antigen design**

*We thank the reviewer for their observations and have revised the manuscript to more explicitly link the statistical findings to the antigen design rationale. The analysis showed that HBV genotype A accounts for more than twice the proportion of circulating sequences compared with genotype D, whereas genotype D exhibits a relatively higher proportion of sequences harboring EMAVs. These contrasting quantitative patterns informed the distinct compositions of MeRPYS1 and MeRPYS2.*

*MeRPYS1 was designed to explore a broader mutation-inclusive architecture by integrating multiple EMAV-containing “a” determinant segments from both genotypes, reflecting both genotype prevalence and EMAV burden. In contrast, MeRPYS2 emphasizes wild-type genotype A sequences in proportion to its higher circulation frequency, while incorporating only a single, highly prevalent EMAV (P127T) from genotype D. This design represents a more conservative mutation-inclusion strategy.*

*We have revised the Results and Discussion to clarify that these constructs reflect alternative, hypothesis-generating design strategies derived from genotype prevalence and EMAV distribution statistics, rather than functionally optimized antigens.*

**4. Epitope prediction redundancy inflates perceived immunogenicity**

The repeated duplication of identical or near-identical epitopes is presented as beneficial, but:

- Epitope repetition does not guarantee improved immunogenicity
- Repetition can cause epitope masking, misfolding, or immune tolerance

The manuscript mentions this limitation briefly, but still implicitly treats duplication as advantageous.

**Recommendation:** Strengthen critical discussion:

- Explicitly state that epitope duplication is speculative
- Cite studies where repetition failed or reduced immunogenicity

**Response to Comment 4: Epitope prediction redundancy**

*We thank the reviewer for this important comment. We have revised the manuscript to explicitly state that the repeated inclusion of identical or near-identical epitopes is a* ***speculative design feature****, intended to explore potential effects on epitope presentation rather than to imply enhanced immunogenicity. The Discussion now highlights that epitope repetition does not guarantee improved immune responses.*

**5. Molecular docking and MD simulation limitations are underemphasized**

**Concerns:**

- Only one antibody structure (PDB: 6VJT) was used
- MD simulations were run once per complex, without replicas
- Binding free energy was not calculated (e.g., MM-PBSA/MM-GBSA)

**Recommendation: Either:**

- Add justification for these limitations or
- Explicitly acknowledge that docking + MD are qualitative, not quantitative

**Response to comment 5: Molecular docking and MD simulation limitations**

*We thank the reviewer for their observations. We have clarified in the manuscript that the docking and MD simulations are* ***qualitative, hypothesis-generating analyses*** *rather than quantitative measures of binding affinity. Only a single antibody structure was used, and no replicas or binding free energy calculations were performed. These limitations are now explicitly acknowledged in the limitation section to prevent over-interpretation.*

**6. Diagnostic relevance remains hypothetical**

The manuscript repeatedly frames the antigens as candidates for diagnostic assay development, but:

- No comparison is made to existing commercial HBsAg antigens
- No sensitivity/specificity modeling is attempted

**Recommendation:** Tone down diagnostic claims or add a short section outlining:

- How these antigens would outperform current kits
- What specific diagnostic formats are envisioned (ELISA, lateral flow, etc.)

**Response to Comment 6: Diagnostic relevance**

*We thank the reviewer for this important observation. We agree that, in the absence of experimental evaluation, any discussion of diagnostic applications should be framed cautiously. We have revised the manuscript to clarify that the designed antigens are hypothesis-generating candidates for potential diagnostic use, rather than validated reagents. We also added a statement indicating that future work could explore these constructs in specific diagnostic formats, such as ELISA or lateral flow assays, to assess their practical utility and performance relative to established kits.*

**Minor Comments (Should be addressed)**

- Language and clarity Numerous grammatical errors (e.g., “excremental validation” → experimental validation)
- Inconsistent spacing, punctuation, and hyphenation
- Figure density Some figures (especially MD plots) are excessive for PLOS ONE
- Consider moving secondary MD plots to Supporting Information
- Methods reproducibility Random seed handling in MD simulations should be more clearly described
- Provide exact software versions consistently (some are missing)
- Ethics statement Although sequence data are public, explicitly state that no new human data were generated
- References Some citations supporting epitope prediction accuracy are outdated
- Consider adding recent benchmarking studies (2022–2024)

**Response to Minor Comments**

*We thank the reviewer for these constructive comments. The manuscript has undergone comprehensive language editing to correct grammatical errors and to standardize spacing, punctuation, and hyphenation throughout. Figure content has been streamlined, and secondary MD simulation plots have been moved to the Supporting Information to improve clarity and alignment with PLOS ONE guidelines.*

*Methods sections were revised to enhance reproducibility, including explicit reporting of all software versions and clarification of random seed handling in MD simulations. An ethics statement has been added to clearly state that only publicly available sequence data were used and that no new human samples were generated. Finally, outdated references supporting epitope prediction were updated, and recent benchmarking studies (2022–2024) have been incorporated where appropriate.*

**Overall Recommendation**

**Minor Revision**

**Rationale:** The study is technically solid, regionally relevant, and suitable for PLOS ONE if claims are appropriately restrained and methodological justifications strengthened. The manuscript currently reads closer to a computational validation paper than a diagnostic development study, and this distinction must be made explicit.

**Response to Overall Recommendation**

*We appreciate this assessment. In response, the manuscript has been revised to clearly distinguish its scope as a hypothesis-generating, computational investigation rather than a diagnostic development or validation study. Claims regarding diagnostic applicability have been restrained, and language implying functional or clinical efficacy has been removed. Methodological justifications have been strengthened to emphasize regional sequence prioritization and exploratory antigen design, consistent with the aims and standards of PLOS ONE.*
